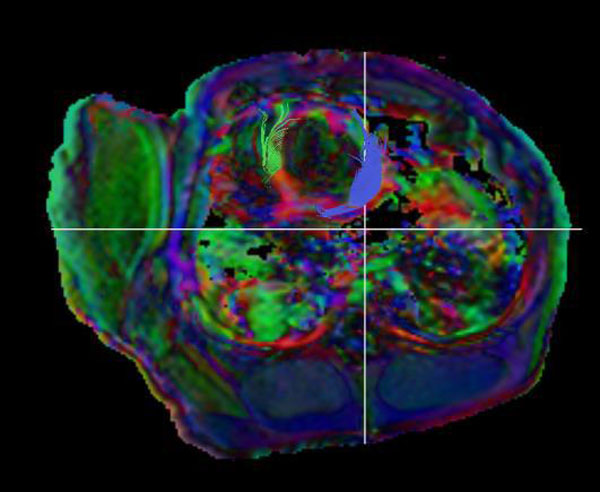# Feasibility of free-breathing diffusion tensor imaging in porcine acute myocardial infarction model

**DOI:** 10.1186/1532-429X-16-S1-P72

**Published:** 2014-01-16

**Authors:** Joon-Won Kang, Seong Hoon Choi, Joon Ho Choi, Yoonyoung Choi, So Youn Shin, Jong Chun Park, Tae-Hwan Lim

**Affiliations:** 1 Department of Radiology, Asan Medical Center, Seoul, Korea, Republic of; 2 Department of Radiology, Ulsan University Hospital, Ulsan, Korea, Republic of

## Background

In vivo DT-MRI is challenging because the motion of the heart and respiration influence the parameters of diffusion tensor imaging, and the most of in vivo DT-MRI is performed under breath-hold. The purpose of this study was to evaluate the feasibility of in vivo DT-MRI without breath-hold with regard to changes in direction-dependent water diffusivity reflecting alterations in tissue integrity such as apparent diffusion coefficients (ADC), fractional anisotropy (FA), and fiber length.

## Methods

Acute myocardial infarction (AMI) was induced by ligation of mid segment of left anterior descending coronary artery (LAD) in sixteen pigs. DT-MRI using a SENSE-based echo-planar imaging technique was acquired using a 1.5-tesla MR scanner with free-breathing state using navigator sequence. With a b-value of 300 s/mm2, the diffusion tensor images were obtained for 6 diffusion-sensitizing gradient directions at infarcted zone at the mid-ventricular level. Image quality of the acquired DTI was evaluated using a 3-grade system; good, fair, and poor. The ADC, FA, and the fiber length were measured for quantitative analysis. The difference of parameters of DT-MRI was evaluated using Wilcoxon signed rank test. Intraobserver agreement of ADC and FA was evaluated using Bland-Altman plots.

## Results

A total of 7 DTI-MRI's were acquired. Image quality was good in 3 pigs, fair in 2 pigs, and poor in 2 pigs. The acquisition time for DT-MRI was 8 ± 1.5 minutes. The infarct zone showed significantly increased ADC than that of the remote zone (8.097 ± 3.741 × 10^-3 ^mm^2^/sec versus 5.894 ± 2.985 × 10^-3 ^mm^2^/sec, P = 0.018). The FA of the infract zone was seen to be also significantly lower than that of remote zone (0.393 ± 0.972 versus 0.485 ± 0.145, P = 0.018). The fiber length in the infarct zone was seen to be significantly shorter than the remote zone (17.57 ± 5.46 mm versus 24.84 ± 9.79 mm, P = 0.018). The difference between the two measurements of ADC and FA didn't show systemic error (P > 0.05)

## Conclusions

In vivo DT-MRI's of post-infarct myocardium with fair or good image quality can be acquired and the results correlated well with those of ex-vivo and breath-hold studies in the literature. This technique may help one understand structural correlates of functional remodeling after infarction especially in the patients who cannot hold one's breath.

## Funding

This work was supported by the National Research Foundation of Korea(NRF) Grant funded by the Korean Government.

**Figure 1:**